# Predictive Accuracy of Serum N-Terminal Pro-B-Type Natriuretic Peptide Alone and in Combination with Respiratory Function Tests to Identify Systemic Sclerosis-Associated Pulmonary Arterial Hypertension (SSc-PAH)

**DOI:** 10.3390/diagnostics16142254

**Published:** 2026-07-19

**Authors:** Zoe Brown, Dylan Hansen, Laura Ross, Wendy Stevens, Kathleen Morrisroe, Alannah Quinlivan, Maryam Tabesh, Gene-Siew Ngian, Diane Apostolopoulos, Joanne Sahhar, Jennifer G. Walker, Lauren Host, Susanna Proudman, Mandana Nikpour

**Affiliations:** 1 Department of Rheumatology, St Vincent’s Hospital, Melbourne, VIC 3065, Australia; 2 Department of Medicine at St Vincent’s Hospital, The University of Melbourne, Melbourne, VIC 3065, Australia; 3 Department of Rheumatology, Monash Health, Clayton, VIC 3168, Australia; 4 Department of Medicine, Monash University, Clayton, VIC 3168, Australia; 5 Rheumatology Unit, Flinders Medical Centre, Bedford Park, SA 5042, Australia; 6 Immunology, Allergy and Arthritis Department, Flinders University, Bedford Park, SA 5042, Australia; 7 Fiona Stanley Hospital, Perth, WA 6150, Australia; 8 Rheumatology Unit, Royal Adelaide Hospital, Adelaide, SA 5000, Australia; 9 Department of Medicine, The University of Adelaide, Adelaide, SA 5000, Australia; 10 Sydney Musculoskeletal Research Centre and School of Public Health, University of Sydney, Sydney, NSW 2050, Australia; 11 Department of Rheumatology, Royal Prince Alfred Hospital, Sydney, NSW 2050, Australia

**Keywords:** systemic sclerosis, pulmonary arterial hypertension, screening

## Abstract

**Background/Objectives**: Pulmonary arterial hypertension (PAH) occurs in >12% of systemic sclerosis (SSc) patients and has a high mortality. Annual screening for all SSc patients is the standard of care and can include spirometry (RFT), serum N-terminal pro-B-type natriuretic peptide (NT-proBNP) and echocardiography (TTE). We sought to evaluate the accuracy of NT-proBNP alone to screen for SSc-PAH. **Methods**: We defined groups as low, intermediate and high risk of PAH according to RFT, TTE and RHC (right heart catheter) parameters. NT-proBNP thresholds predictive of risk group were defined, and ROC curves were used to evaluate the predictive accuracy of the calculated cut-points to diagnose SSc-PAH within the following 12 months. **Results**: A total of 820 SSc patients had NT-proBNP recorded 12 months prior to RHC, or if no RHC had been performed, within 12 months of the most recently recorded annual visit. The majority (335, 40.85%) were assigned to low risk of PAH, 189 (23.05%) to intermediate risk and 176 (21.46%) to high risk. NT-proBNP level < 122.9 ng/L defined the low-risk group, 122.9–186.6 ng/L defined the intermediate risk and >186.6 ng/L defined the high risk. NT-proBNP ≥210 ng/L with RFT (the ‘ASIG algorithm’) had the highest predictive accuracy, with sensitivity of 86.75% (77.52–93.19%), specificity of 60.71% (58.52–62.87%), PPV of 8.47% (6.69–10.55%), NPV of 99.09% (98.38–99.55%) and AUC of 0.74 (0.70–0.78). NT-proBNP ≥ 210 ng/L alone had sensitivity of 67.05% (56.21–76.70), specificity of 71.91% (70.03–73.72), PPV of 8.25% (6.34–10.52), NPV of 98.30% (97.57–98.86) and AUC of 0.69 (0.64–0.74). NT-proBNP thresholds associated with the three defined risk groups performed similarly overall. **Conclusions:** NT-proBNP alone, compared to the ASIG algorithm, had a slight reduction in NPV and AUC to predict PAH within 12 months. When NT-proBNP is ≥210 ng/L, the NPV remains high (98.0%).

## 1. Introduction

Pulmonary arterial hypertension (PAH) occurs in over 12% of patients with systemic sclerosis (scleroderma; SSc) and is one of the leading causes of mortality [1]. The prevalence of this SSc manifestation and access to effective therapy necessitate systematic screening of asymptomatic SSc patients in order to make an early diagnosis of PAH. Early diagnosis and treatment of SSc-PAH has been associated with improved survival [2,3,4].

International PAH management guidelines recommend annual screening for all asymptomatic SSc patients using composite screening algorithms, which include respiratory function tests (RFTs), and serum N-terminal pro-B-type natriuretic peptide (NT-proBNP) prior to transthoracic echocardiography (TTE), such as the Australian Scleroderma Interest Group (ASIG) Algorithm [5]. Multimodal screening, with algorithms such as the ASIG and DETECT Algorithms, has been shown to be more accurate compared to TTE alone [5,6,7].

However, there are recognized barriers to accessing RFT, particularly RFT including measurement of diffusion capacity, which has highlighted the need to understand the utility of screening investigations in isolation [8]. For example, access to respiratory diagnostic services, particularly RFT, was negatively affected during the COVID-19 pandemic, during which time consensus guidelines recommended the triaging of referrals based on urgency and deferral of routine testing [9].

We sought to evaluate the effectiveness of NT-proBNP alone compared to NT-proBNP combined with RFTs (ASIG screening algorithm) to identify patients at risk of a diagnosis of PAH within 12 months. Comparison to the ASIG algorithm was selected, as the ASIG algorithm is increasingly applied to patients enrolled in the Australian Scleroderma Cohort Study (ASCS) and requires only two variables, compared to six required to complete the DETECT algorithm.

We hypothesize that NT-proBNP alone may perform with similar predictive ability to the ASIG algorithm as a screening test and therefore be a reasonable substitute for screening SSc patients for PAH when unable to complete RFT.

## 2. Materials and Methods

Study design and patient population: We performed analyses of prospectively collected data from SSc patients undergoing annual PAH screening. We included SSc patients fulfilling the ACR/EULAR classification criteria for SSc and/or mixed connective tissue disease (MCTD) and enrolled in the Australian Scleroderma Cohort Study (ASCS) [10,11]. Both patients with SSc and MCTD are at risk of developing PAH [12].

The ASCS began in 2007 as a multicenter national registry, in which clinical and diagnostic data are recorded annually for patients with SSc attending for prospective screening for the cardiopulmonary complications of SSc, including PAH. The ASCS has Human Research and Ethics Committee approval for all study sites. Informed consent for participation was obtained from all subjects involved in the study.

Whilst the ASCS has been screening SSc patients annually for the early diagnosis of PAH according to contemporaneous recommended guidelines from 2007 (using clinical examination, TTE and RFT), serum NT-proBNP has only been accessible with Medicare Benefit Scheme reimbursement from 01 November 2023. Access to NT-proBNP prior to this date was infrequently available at registry sites, being available regularly at one study site in Adelaide (South Australia) from 2015, and in the majority of study sites, becoming available from November 2023. Serum NT-proBNP is measured using the Roche electrochemiluminescence immunoassay for quantitative determination.

To determine thresholds of serum NT-proBNP as a biomarker for risk of PAH among SSc patients attending for annual screening visits with an NT-proBNP recorded, we defined three risk groups: low, intermediate and high risk of a diagnosis of PAH. The definition of risk groups was based upon the European Society of Cardiology/European Respiratory Society (ESC/ERS) 2022 guidelines for the diagnosis of pulmonary hypertension (PH) interpretation of respiratory RFT, and TTE parameters [5].

**Figure 1:**
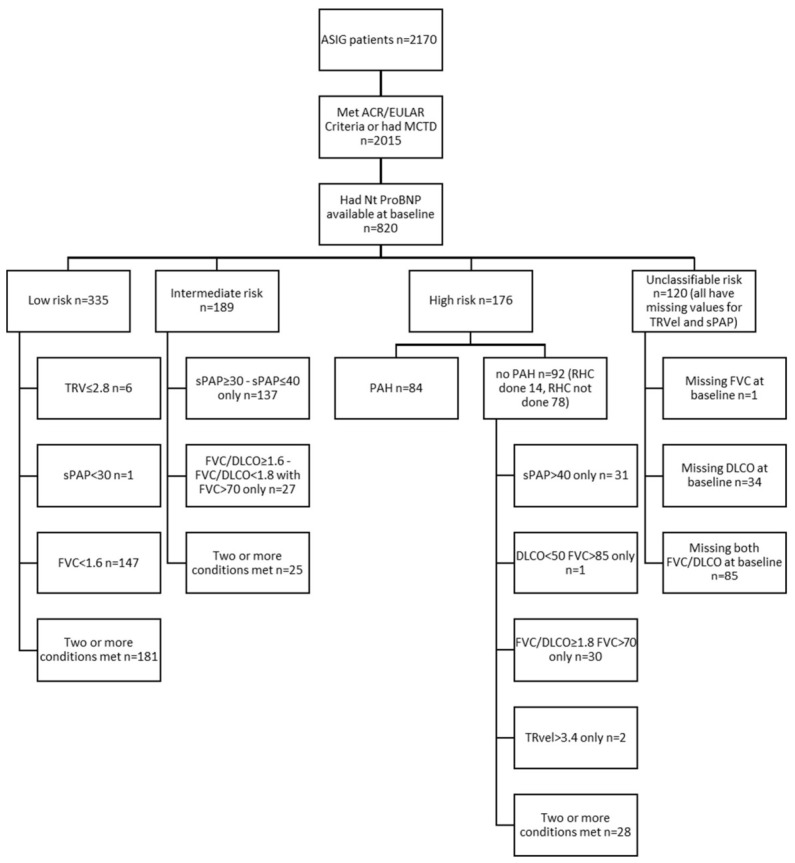
Flowchart of subject numbers through the process of assignment of risk group. ASIG, Australian Scleroderma Interest Group; ACR/EULAR, American College of Rheumatology/European League Against Rheumatism; MCTD, mixed connective tissue disease; TRVel, tricuspid regurgitant jet velocity; sPAP, systolic pulmonary artery pressure; PAH, pulmonary arterial hypertension; RHC, right heart catheter; FVC, forced vital capacity; DLCO, diffusion capacity for carbon monoxide.

Patients were defined as high risk of having PAH if they had right heart catheter (RHC)-confirmed PAH meeting the most recent definition (mean pulmonary artery pressure (mPAP) > 20 mmHg, pulmonary arterial wedge pressure (PAWP) ≤ 15 mmHg and pulmonary vascular resistance (PVR) > 2 Woods Units (WU)) (Figure 1) [5]. As this registry predated the current definition, patients with PAH defined as mPAP ≥ 25 mmHg, PAWP ≤ 15 mmHg and a missing PVR were also defined as high risk [5]. Patients who had not had clinical indications to undergo an RHC were defined as high risk of PAH in the presence of TTE systolic pulmonary artery pressure (sPAP) > 40 mmHg or tricuspid regurgitant jet velocity (TRV) > 3.4 m/s or by RFT parameters, diffusion capacity for carbon monoxide (DLCO) < 50%, or forced vital capacity%/DLCO ratio (FVC/DLCO) ratio of ≥1.8 and FVC > 70%. Therefore, it was possible to be classified as high risk of PAH if there were missing values for sPAP or TRV or RFT parameters. Those with missing values for sPAP and TRV are reported as unclassifiable risk.

The intermediate-risk group was defined by TTE parameters of sPAP ≥ 30 and ≤40 mmHg, or TRV > 2.8 and ≤3.4 m/s or RFT parameters FVC/DLCO ≥ 1.6 to <1.8 with FVC >70%. Patients were assigned to the low-risk group if TTE parameters were TRV ≤ 2.8 m/s, or sPAP < 30 mmHg or by RFT parameters FVC/DLCO < 1.6.

As NT-proBNP and the ASIG algorithm are intended as screening tests and not as diagnostic tests and are therefore used to identify patients with a high likelihood of PAH, we did not require patients to meet the gold standard diagnostic definition of PAH to be included in these risk groups [5].

Baseline was defined as the visit at which RHC confirmed PAH, or for those who had not had a RHC the most recently recorded visit.

Statistical analyses: Patients with an NT-proBNP recorded in the whole cohort, low-, intermediate- and high-risk groups were compared and presented with a *p*-value calculated by Chi-square test. For normally distributed continuous variables, the mean (standard deviation) is presented with a *p*-value calculated by ANOVA. For non-normally distributed continuous variables, the median (interquartile range) is presented with a *p*-value calculated by Kruskal–Wallis equality-of-populations rank test. A box plot of serum NT-proBNP natural log-transformed by risk group was created.

The thresholds for serum NT-proBNP levels predictive of risk group assignment were derived using the Liu method of maximizing the area under the curve (AUC) for the low-risk or high-risk group with the intermediate group being those who fall between the two points [13].

Receiver operator characteristic (ROC) curve analysis was used for serum NT-proBNP to determine optimal cut-points to predict a diagnosis of PAH (defined according to the RHC-confirmed definitions described above) within 12 months following the test being performed. Patients who did not undergo RHC or did not meet the RHC-confirmed definition of PAH were defined as negative for PAH. Other cut-points were also analyzed, including the Australian Scleroderma Interest Group (ASIG), ‘component A’, NT-proBNP ≥ 210 ng/L, combined with RFT parameters ‘component B’, NT-proBNP ≥ 210 ng/L alone; and the cut-points derived based on low-, intermediate- and high-risk groups and presented as sensitivity, specificity negative and positive predictive values and AUC.

We undertook a sensitivity analysis by performing ROC curve analysis according to the methods above, though only including those with RHC-confirmed PAH and serum NT-proBNP availability from 1 January 2016, as this is the date patients enrolled in the ASCS in South Australia gained access to screening with NT-proBNP.

## 3. Results

As of 14 June 2024, there were 2170 patients enrolled in ASCS, and 2015 met classification criteria for ACR/EULAR SSc and/or MCTD. Of these, 820 (40.69%) had a serum NT-proBNP recorded 12 months prior to a right heart catheter (RHC), or if no RHC had been performed, serum NT-proBNP within 12 months of the most recently recorded annual visit.

The majority of patients with an NT-proBNP result (335, 40.85%) were assigned to low risk of PAH, 189 (23.05%) to intermediate risk and 176 (21.46%) to high risk of PAH. There were 120 participants (14.63%) in whom a risk category was unable to be assigned due to missing or unrecordable TRV and sPAP; and RFT variables were missing in 85 participants. A flowchart of risk-group assignment is shown in Figure 1.

Characteristics of those in each determined risk group were compared and shown in Table 1. Among 700 patients able to be classified into risk groups, there were 71 cases of PAH (10.1%). Those deemed to be at low risk of PAH were younger at recruitment to ASCS (age, 53.99 years (44.62–61.12)) compared to those in intermediate- and high-risk groups, respectively (60.34 years (52.03–68.04) vs. 63.86 years (56.34–72.01), *p* < 0.001). There were no significant differences between risk groups with respect to sex, race or disease duration or autoantibody profile at risk assignment.

Those classified at high risk of a diagnosis of PAH (including those with RHC-confirmed PAH) based upon RHC, TTE and RFT parameters had more frequent ILD classified as severe on HRCT compared to intermediate. To compare those with PAH to those without, characteristics at the time of RHC or most recent review are shown in Supplementary Table S1.

RFT parameters were significantly different between risk groups, noting they do form part of the group-allocation criteria. FVC% was lowest in those at high risk compared to intermediate- and low-risk groups, respectively (90.00% (72.00–107.5) vs. 98.50% (83.00–112.00) vs. 96.00% (82.00–107.00), *p* = 0.025. DLCO% was also lowest among the high-risk group, 50.00% (39.00–63.00) vs. 74.00% (62.00–83.86) and 82.37% (69.14–94.00), *p* < 0.001 (Table 1).

WHO functional class was significantly different between risk groups, with the majority in low risk classified as WHO functional class I (217 (68.2%) vs. 89 (51.4%) and 27 (16.05%), *p* < 0.001, in intermediate and high-risk groups, respectively. More participants assigned to high-risk groups had cardiovascular disease (CVD) comorbidities, including systemic hypertension and a history of angina or acute myocardial infarction (AMI), than those in lower-risk groups. Systemic hypertension was recorded in 89 (51.1%) of those in high risk compared to 92 (48.7%) and 130 (38.9%), *p* = 0.013 in intermediate and low-risk groups, respectively. Angina/AMI had occurred in 21 (12.1%) of those in the high-risk group compared to 17 (9.0%) and 16 (4.8%), *p* = 0.010, in the intermediate and low-risk groups, respectively (Table 1).

PAH prognostic indicators were significantly different among risk groups. Six-minute walk distance (6MWD) was lowest among those in the high-risk group compared to intermediate and low-risk groups (379.00 m (306.00–445.00) vs. 490.00 m (400.00–550.00) and 491.50 m (435.00–550.00), *p* < 0.001). NT-proBNP was highest in the high-risk group (272.72 ng/L (123.00–722.42) vs. 142.00 (80.85–274.00) vs. 94.55 (55.82–179.00), *p* < 0.001). Serum uric acid was highest in the high-risk group (0.34 mmol/L (0.26–0.41) vs. 0.30 mmol/L (0.24–0.36) vs. 0.28 (0.22–0.33), *p* < 0.001), Table 1.

TTE parameters were also significantly different amongst risk groups, noting that TRV and sPAP were part of the risk-assignment criteria. Right atrial area (RAA) was largest among those in the high-risk group (17.00 cm^2^ (14.80–20.00) vs. 15.00 cm^2^ (13.00–17.00) vs. 14.00 cm^2^ (12.00–16.00), *p* < 0.001) compared to intermediate and low-risk groups respectively. TRV was absent in 30 (17.05%) of those in the high-risk group, 28 (14.8%) in the intermediate group and 168 (50.1%) in the low-risk group (*p* < 0.001). More patients in the high-risk group had a pericardial effusion by TTE at the time of RHC or most recently recorded review if no RHC had been performed (16 (9.4%) vs. 6 (3.3%) vs. 6 (2.1%), *p* = 0.001).

RHC parameters were significantly different among risk groups, although noting that a diagnosis of PAH resulted in assignment to the high-risk group. In particular, mPAP was higher in the high-risk group compared to the intermediate- and low-risk groups (29.00 mmHg (25.00–35.00) vs. 21.00 mmHg (18.00–23.00) vs. 14.00 (13.00–17.00), *p* < 0.001), and PVR was highest in the high-risk group (3.20 WU (2.39–5.18) vs. 1.53 (0.80–1.80) vs. 1.74 (1.00–1.90), *p* < 0.001.

Using the Liu method of maximizing the AUC for the low-risk or high-risk group, with the intermediate group being those who fall between the two points, a serum NT-proBNP level < 122.9 ng/L was found to associate with assignment to low risk, NT-proBNP 122.9–186.6 ng/L to intermediate risk and >186.6 ng/L with high risk [13].

Further ROC curve analysis of NT-proBNP thresholds was undertaken and presented as sensitivity, specificity, positive and negative predictive value (PPV and NPV, respectively), and AUC with 95% Confidence Interval (CI) for identifying PAH according to RHC undertaken within 12 months (Table 2). Therefore, only patients who had undergone RHC were able to be included.

Serum NT-proBNP was evaluated as part of the ASIG algorithm (as ‘component A’ used in combination with the RFT parameters as ‘component B’). NT-proBNP thresholds associated with each risk group were determined by the Liu method, as described above.

Serum NT-proBNP ≥ 210 ng/L used in combination with RFT, as in the ASIG algorithm, had the highest predictive accuracy with sensitivity of 86.75% (77.52–93.19%), specificity of 60.71% (58.52–62.87%), PPV of 8.47% (6.69–10.55%), NPV of 99.09% (98.38–99.55%), and AUC of 0.74 (0.70–0.78). NT-proBNP ≥210 ng/L alone had sensitivity of 67.05% (56.21–76.70), specificity of 71.91% (70.03–73.72), PPV of 8.25% (6.34–10.52), NPV of 98.30% (97.57–98.86) and AUC of 0.69 (0.64–0.74).

Serum NT-proBNP thresholds associated with the three risk groups as previously defined, performed similarly overall. NT-proBNP > 122.9 ng/L associated with intermediate risk of PAH had sensitivity of 79.55% (69.61–87.40%), specificity of 52.55% (50.50–54.59%), PPV of 5.94% (4.66–7.45%), NPV of 98.55% (97.72–99.14%), and AUC of 0.66 (0.62–0.70).

Sensitivity analysis revealed that the ASIG had the highest predictive accuracy, with sensitivity of 100.00% ((84.56–100.00%) to identify PAH, NPV 100.00% (99.58–100.00%) and AUC 0.82 (0.81–0.83). Serum NT-proBNP alone had sensitivity of 77.27% (54.63–92.18%), NPV of 99.60% (99.07–99.87%) and AUC of 0.76 (0.67–0.85) (Supplementary Table S2).

**Figure 2:**
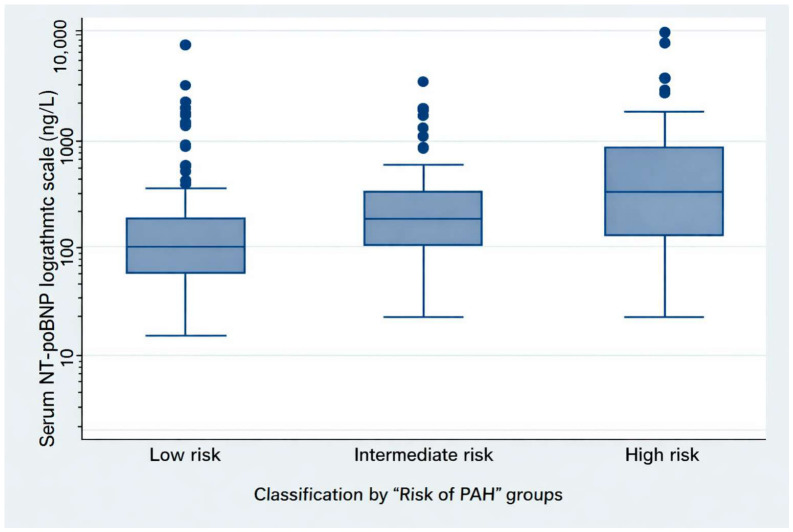
Box plot of logarithmic scale serum NT-proBNP levels by risk group.

The distribution of serum NT-proBNP levels natural log transformed between risk groups is displayed in Figure 2 and demonstrates that mean serum NT-proBNP levels were highest in those defined at high risk of PAH or having PAH.

Serum NT-proBNP level was evaluated as a continuous variable to predict a diagnosis of PAH (RHC-confirmed) within 12 months was evaluated by ROC curve analysis (Supplementary Figure S1), with an area under the curve (AUC) of 0.7334.

## 4. Discussion

In this cohort of SSc patients attending for annual screening for PAH, low-, intermediate- and high-risk groups were defined by risk of PAH based on RHC, TTE and RFT parameters known to be associated with a diagnosis of PAH; additionally, those with RHC-proven PAH were included in the high-risk group [5]. Cut-points of NT-proBNP to define the risk groups were low risk, <122.9 ng/L; intermediate risk, 122.9–186.6 ng/L; and high risk, >186.6 ng/L [13].

Of the included 820 SSc patients with an NT-proBNP result available, 120 (14.6%) had absent TRV and were therefore unable to be classified into a risk group. Of those who were able to be classified on the basis of other available parameters, TRV was missing or unrecorded in 17.0% of those with RHC-confirmed PAH or deemed at high risk of PAH, and in 50.1% of those at low risk. Absent TRV jet on echocardiography has been reported with similar frequencies to our analysis among those without PAH in other SSc cohorts and in 7% of those with PAH [14]. As a result of this, composite screening algorithms, which include multimodal parameters such as serum NT-proBNP and RFT as an initial step prior to echocardiography, have been shown to be more accurate for screening for SSc-PAH [6,7,15].

Serum NT-proBNP ≥ 210 ng/L, the threshold used to define a positive component in the ASIG algorithm, had an AUC of 0.69 (0.64–0.74) compared to an AUC 0.74 (0.70–0.78) for the ASIG algorithm; therefore, NT-proBNP alone performed comparatively well, with only a slight reduction in specificity and NPV compared to the combination of NT-proBNP and RFT in the ASIG algorithm. It should be noted that the sensitivity of NT-proBNP alone (67%) is reduced compared to the ASIG algorithm, and in cases of clinical concern, patients should be referred for further evaluation and investigation for possible PAH.

The predictive accuracy of the ASIG algorithm to detect PAH within 12 months in this analysis is similar to that shown in previous analyses [6,7,15]. The ASIG algorithm in this analysis had high NPV 99.0% (98.38–99.55%) to rule out PAH in those with a negative result, which is a desirable performance characteristic in a screening algorithm.

Overall, this is reassuring evidence that in circumstances in which patients may present with serum NT-proBNP alone to screen for PAH, for example, patients living remotely or regionally, NT-proBNP alone has a high NPV to rule out those at high risk of having PAH. Using the NT-proBNP threshold of ≥210 ng/L, as in the ASIG algorithm, optimizes the AUC to predict PAH within 12 months.

The limitations of this analysis are that not all patients in this cohort underwent the gold-standard diagnostic test to identify or rule out PAH, RHC. This impacts the homogeneity of patients included in the classified risk groups and confidence in the accuracy of excluding those with PAH in the moderate- and low-risk groups. However, RHC is an invasive procedure and serious adverse events are reported in 1.1% when performed in PH centers, and it is therefore unfeasible and unethical in all patients, in the absence of other clinical indicators to proceed [5]. Therefore, the NPV in this analysis is reflective of the test characteristic evaluated in an enriched cohort who were selected for referral for RHC and therefore were at high risk of PAH.

Further evaluation of NT-proBNP alone in bigger and more diverse cohorts would be needed to further inform the generalizability of these findings. As the NT-proBNP thresholds were defined in the ASCS, external validation of the predictive accuracy of NT-proBNP according to these thresholds is necessary to more accurately interpret these findings.

## 5. Conclusions

Annual screening using multimodal screening algorithms for all patients with SSc in order to make an early diagnosis of PAH is the standard of care and is associated with improved survival [2,3,4]. Serum NT-proBNP alone was associated with a slight reduction in NPV and AUC to predict PAH within 12 months, compared to a complete ASIG algorithm screen combined with RFT parameters. NT-proBNP alone with a threshold of ≥ 210 ng/L, as in the ASIG algorithm, maintains a high NPV (99.0%), meaning those with a negative screening test result have a low likelihood of having PAH. The interpretation of serum NT-proBNP alone for screening for SSc-PAH requires further exploration in more diverse SSc populations, though this analysis provides some reassurance that this parameter alone may be helpful to rule out those at high risk of PAH.

## Figures and Tables

**Table 1:** Patient characteristics by assigned risk group (NB-only patients with a recorded NT-proBNP included and those unclassifiable excluded).

Variable	All Patients (*n* = 700)	High Risk (*n* = 176)	Intermediate Risk (*n* = 189)	Low Risk (*n* = 335)	*p*-Value
Age at recruitment into database (years)	58.13 (49.12–66.23)	63.86 (56.34–72.01)	60.34 (52.03–68.04)	53.99 (44.62–61.12)	<0.001
Sex Male	94 (13.4%)	27 (15.4%)	20 (10.6%)	47 (14.0%)	0.364
Female	605 (86.6%)	148 (84.6%)	169 (89.4%)	288 (86.0%)	0.364
Race Aboriginal–Islander	6 (0.9%)	4 (2.4%)	0 (0.0%)	2 (0.6%)	0.282
Caucasian	611 (92.4%)	157 (93.5%)	171 (95.0%)	283 (90.4%)	
Asian	35 (5.3%)	6 (3.6%)	7 (3.9%)	22 (7.0%)	
Hispanic	3 (0.5%)	0 (0.0%)	1 (0.6%)	2 (0.6%)	
Other	5 (0.8%)	1 (0.6%)	1 (0.6%)	3 (1.0%)	
Polynesian	0 (0.0%)	0 (0.0%)	0 (0.0%)	0 (0.0%)	
Middle Eastern	1 (0.2%)	0 (0.0%)	0 (0.0%)	1 (0.3%)	
Disease duration at recruitment * (years)	6.74 (2.09–15.49)	9.82 (3.43–20.29)	6.44 (2.28–15.10)	5.53 (1.67–13.70)	<0.001
Disease duration to time of RHC/most recent review if no RHC * (years)	12.67 (6.00–20.81)	13.83 (7.16–22.92)	12.12 (5.43–20.84)	12.39 (6.20–19.70)	0.312
PAH (mPAP ≥ 20 mmHg, PAWP ≤ 15 mmHg, PVR > 2 WU)	71 (10.1%)	71 (40.3%)	0 (0.0%)	0 (0.0%)	<0.001
NT-proBNP cut-off #					
NT-proBNP < 122.9 ng/L (low-risk group)	333 (47.6%)	44 (25.0%)	80 (42.3%)	209 (62.4%)	<0.001
NT-proBNP 122.9–186.6 ng/L (intermediate-risk group)	103 (14.7%)	20 (11.4%)	34 (18.0%)	49 (14.6%)	
NT-proBNP > 186.6 ng/L (high-risk group)	264 (37.7%)	112 (63.6%)	75 (39.7%)	77 (23.0%)	
Autoantibody profile (ever)					
ANA positive	669 (96.1%)	169 (96.0%)	179 (94.7%)	321 (97.0%)	0.434
ANA nucleolar	154 (22.2%)	42 (23.9%)	40 (21.3%)	72 (21.8%)	0.818
ANA homogenous	137 (19.8%)	29 (16.5%)	39 (20.7%)	69 (21.0%)	0.446
ANA centromere	312 (44.9%)	82 (46.6%)	90 (47.6%)	140 (42.4%)	0.453
ENA Scl-70	101 (14.6%)	20 (11.4%)	31 (16.6%)	50 (15.2%)	0.354
RNA polymerase III	95 (16.4%)	24 (16.3%)	22 (14.4%)	49 (17.4%)	0.713
ANCA	147 (22.1%)	37 (21.9%)	33 (18.1%)	77 (24.5%)	0.254
Anti-MPO	28 (4.3%)	6 (3.6%)	4 (2.2%)	18 (5.8%)	0.148
Anti-PR3	27 (4.1%)	7 (4.2%)	6 (3.3%)	14 (4.5%)	0.812
Anti-CCP	12 (3.5%)	2 (2.5%)	2 (2.7%)	8 (4.2%)	0.719
Anti-B2 glycoprotein	43 (33.9%)	10 (22.2%)	17 (42.5%)	16 (38.1%)	0.111
Anti-Cardiolipin IgG	54 (40.9%)	17 (35.4%)	14 (34.1%)	23 (53.5%)	0.123
Lupus anticoagulant	21 (3.4%)	6 (3.7%)	5 (3.1%)	10 (3.3%)	0.950
Anti-dsDNA	73 (11.5%)	13 (8.0%)	19 (11.8%)	41 (13.1%)	0.251
ENA Jo-1	2 (0.3%)	0 (0.0%)	1 (0.5%)	1 (0.3%)	0.637
ENA La	15 (2.2%)	4 (2.3%)	5 (2.7%)	6 (1.8%)	0.808
ENA U1RNP	60 (8.7%)	9 (5.1%)	15 (8.0%)	36 (10.9%)	0.085
ENA Ro52	60 (8.7%)	16 (9.1%)	13 (7.0%)	31 (9.4%)	0.617
ENA Scl/PM	15 (2.2%)	2 (1.1%)	2 (1.1%)	11 (3.3%)	0.134
ENA Sm	22 (3.2%)	2 (1.1%)	5 (2.7%)	15 (4.5%)	0.105
Rheumatoid factor	202 (29.8%)	66 (39.1%)	45 (24.7%)	91 (27.8%)	0.008
ILD on HRCT ever (*n* = 350)	201 (57.4%)	77 (60.2%)	52 (61.9%)	72 (52.2%)	0.268
Highest severity of ILD on HRCT ever (*n* = 189) Mild (<20%)	106 (56.1%)	33 (44.6%)	32 (65.3%)	41 (62.1%)	0.001
Moderate (20–30%)	44 (23.3%)	14 (18.9%)	12 (24.5%)	18 (27.3%)	
Severe (>30%)	39 (20.6%)	27 (36.5%)	5 (10.2%)	7 (10.6%)	
Respiratory function test					
FVC % predicted	96.00 (81.00–109.00)	90.00 (72.00–107.50)	98.50 (83.00–112.00)	96.00 (82.00–107.00)	0.025
corrected DLCO % PAH	72.84 (57.00–87.00)	50.00 (39.00–63.00)	74.00 (62.00–83.86)	82.37 (69.14–94.00)	<0.001
WHO functional class (*n* = 660)					
Class I	333 (50.5%)	27 (16.0%)	89 (51.4%)	217 (68.2%)	<0.001
Class II	199 (30.2%)	55 (32.5%)	64 (37.0%)	80 (25.2%)	
Class III	115 (17.4%)	76 (45.0%)	19 (11.0%)	20 (6.3%)	
Class IV	13 (2.0%)	11 (6.5%)	1 (0.6%)	1 (0.3%)	
Cardiovascular disease risk factors					
Systemic hypertension	311 (44.6%)	89 (51.1%)	92 (48.7%)	130 (38.9%)	0.013
Diabetes	49 (7.1%)	18 (10.4%)	10 (5.4%)	21 (6.3%)	0.141
Angina/acute myocardial infarction	54 (7.8%)	21 (12.1%)	17 (9.0%)	16 (4.8%)	0.010
PAH prognostic indicators					
Six-minute walk distance (m) (*n* = 270)	450.00 (363.00–525.00)	379.00 (306.00–445.00)	490.00 (400.00–550.00)	491.50 (435.00–550.00)	<0.001
NT Pro BNP (ng/L) (*n* = 700)	131.59 (68.79–262.50)	272.72 (123.00–722.42)	142.00 (80.85–274.00)	94.55 (55.82–179.00)	<0.001
Uric acid (mmol/L) (*n* = 298)	0.29 (0.24–0.35)	0.34 (0.26–0.41)	0.30 (0.24–0.36)	0.28 (0.22–0.33)	<0.001
Echocardiographic (TTE) parameters					
RA area (cm^2^)	15.00 (13.00–18.00)	17.00 (14.80–20.00)	15.00 (13.00–17.00)	14.00 (12.00–16.00)	<0.001
TR velocity (m/s)	2.50 (2.30–2.80)	3.19 (2.70–3.40)	2.60 (2.42–2.70)	2.20 (2.10–2.40)	<0.001
TR velocity missing	226 (32.3%)	30 (17.0%)	28 (14.8%)	168 (50.1%)	<0.001
sPAP (mmHg)	31.00 (26.00–38.00)	44.00 (37.00–53.00)	33.00 (30.00–36.00)	25.00 (23.00–27.00)	<0.001
Pericardial effusion (non-trivial)	28 (4.3%)	16 (9.4%)	6 (3.3%)	6 (2.1%)	0.001
Right heart catheterization (RHC) parameters					
RAP (mmHg)	7.00 (5.00–10.00)	8.00 (5.00–10.50)	5.00 (4.00–9.50)	5.50 (4.00–8.00)	0.242
mPAP (mmHg)	28.00 (22.50–34.00)	29.00 (25.00–35.00)	21.00 (18.00–23.00)	14.00 (13.00–17.00)	<0.001
PAWP (mmHg)	11.00 (7.00–14.00)	11.00 (7.50–13.50)	14.00 (8.00–20.00)	8.50 (6.00–12.50)	0.314
PVR (WU)	2.99 (2.14–4.80)	3.20 (2.39–5.18)	1.53 (0.80–1.80)	1.74 (1.00–1.90)	<0.001
Missing PVR (*n* = 71)	11 (15.5%)	11 (15.5%)	0 (.%)	0 (.%)	.
Cardiac output (L/min) (thermodilution)	5.00 (4.00–6.03)	5.01 (4.02–6.12)	4.84 (3.97–5.23)	4.90 (4.20–6.00)	0.922
Cardiac index (L/min/m^2^)	2.89 (2.32–3.17)	2.89 (2.32–3.19)	2.82 (2.65–3.06)	2.61 (2.21–3.10)	0.892
Heart rate (bpm)	73.00 (64.00–76.00)	73.00 (70.00–76.00)	63.50 (54.50–76.00)	67.00 (61.00–73.00)	0.359

NT-proBNP, N-terminal proB-type natriuretic peptide; RHC, right heart catheterization; ANA, antinuclear antibody; ENA, extractable nuclear antigen; Scl-70, anti-Scl-70 antibody (anti-topoisomerase antibody); RNA, ribonucleic acid; ANCA, anti-neutrophil cytoplasmic antibody; MPO, anti-myeloperoxidase antibody; PR3, anti-proteinase 3 antibody; Anti-CCP, anti-cyclic citrullinated antibody; anti-dsDNA, anti-double stranded deoxyribonucleic acid antibody; ILD, interstitial lung disease; HRCT, high-resolution computed tomography of the chest; FVC%, forced vital capacity % predicted; DLCO, diffusion capacity for carbon monoxide % predicted; WHO, World Health Organization; RA, right atrial; TR, tricuspid regurgitant jet; sPAP, systolic pulmonary artery pressure; RAP, mean right atrial pressure; mPAP, mean pulmonary artery pressure; PAWP, pulmonary arterial wedge pressure; PVR, pulmonary vascular resistance. * Disease duration from first non-Raynaud’s phenomenon SSc disease manifestation onset. # NT-proBNP cut-offs determined by Liu et al. method to maximize the AUC for low risk and high risk, with intermediate group falling between.

**Table 2:** Results of ROC curve analysis for NT-proBNP at different cut-points to identify PAH within 12 months.

Variable	Sens	Spec	PPV	NPV	AUC
ASIG screening algorithm (NT-proBNP and/or cDLCO < 70% and FVC/DLCO ≥ 1.8)	86.75% (77.52–93.19%)	60.71% (58.52–62.87%)	8.47% (6.69–10.55%)	99.09% (98.38–99.55%)	0.74 (0.70–0.78)
NT ProBNP ≥ 210 ng/L	67.05% (56.21–76.70%)	71.91% (70.03–73.72%)	8.25% (6.34–10.52%)	98.30% (97.57–98.86%)	0.69 (0.64–0.74)
NT ProBNP > 122.9 ng/L	79.55% (69.61–87.40%)	52.55% (50.50–54.59%)	5.94% (4.66–7.45%)	98.55% (97.72–99.14%)	0.66 (0.62–0.70)
NT ProBNP > 186.6 ng/L	69.32% (58.58–78.71%)	67.54% (65.60–69.44%)	7.45% (5.74–9.46%)	98.32% (97.56–98.89%)	0.68 (0.63–0.73)

ROCs, receiver operator characteristics; NT-proBNP, N-terminal pro-brain-type natriuretic peptide; PAH, pulmonary arterial hypertension; Sens, sensitivity; Spec, specificity; PPV, positive predictive value; NPV, negative predictive value; AUC, area under the curve; cDLCO, corrected diffusion capacity for carbon monoxide % predicted; FVC, forced vital capacity % predicted.

## Data Availability

All clinical data were obtained from the Australian Scleroderma Cohort Study (ASCS) repository and are available under a Data Use Agreement from the respective Human Research and Ethics Committees.

## Supplementary Materials

The following supporting information can be downloaded at https://www.mdpi.com/article/10.3390/diagnostics16142254/s1, Table S1: Patient characteristics of those with PAH and without PAH; Table S2: Results of ROC curve analysis for NT-proBNP at different cut-points to identify PAH within 12 months (inclusion from 1 January 2016); Figure S1: Receiver operating characteristics (ROC) curve for serum NT-proBNP level to predict PAH diagnosis within 12 months (AUC = 0.7334).

## Abbreviations

The following abbreviations are used in this manuscript:

ASCS	Australian Scleroderma Cohort Study
ASIG	Australian Scleroderma Interest Group
CTD	connective tissue disease
DLCO%	diffusion capacity for carbon monoxide % predicted
FEV1%	forced expiratory volume % predicted
FVC%	forced vital capacity % predicted
HRCT	high-resolution computed tomography of the chest
NT-proBNP	N-terminal pro-brain-type natriuretic peptide
PH	pulmonary hypertension
PAH	pulmonary arterial hypertension
RFT	respiratory function test
RHC	right heart catheter
SSc	systemic sclerosis, scleroderma
TTE	transthoracic echocardiography
