# Supplementary material for: Predictive Accuracy of Serum N-Terminal Pro-B-Type Natriuretic Peptide Alone and in Combination with Respiratory Function Tests to Identify Systemic Sclerosis-Associated Pulmonary Arterial Hypertension (SSc-PAH)
Source: Diagnostics (Basel). 2026 Jul 19;16(14):2254. doi: 10.3390/diagnostics16142254 (PMC13408370; doi:10.3390/diagnostics16142254)
Supplement: Supplementary file 1 [file diagnostics-16-02254-s001.zip › diagnostics-4222667-supplementary.pdf]

Supplementary Table S1. Patient characteristics of those with PAH and without PAH

Variable	All patients (n=820)	PAH (n=71)	No PAH (n=749)	p value
**Demographics**				
NTproBNP optimal cut-point for group assignment				
Low risk (<122.9ng/L)	391 (47.7%)	14 (19.7%)	377 (50.3%)	<0.001
Intermediate risk (122.9 - 186.6ng/L)	123 (15.0%)	7 (9.9%)	116 (15.5%)	
High risk (>186.6ng/L)	306 (37.3%)	50 (70.4%)	256 (34.2%)	
Age at recruitment into database (years)	57.39 (48.15-65.00)	62.51 (56.93-71.98)	56.47 (47.01-64.41)	<0.001
Gender - Male	109 (13.3%)	13 (18.3%)	96 (12.8%)	0.194
Gender - Female	710 (86.7%)	58 (81.7%)	652 (87.2%)	
Race				
Aboriginal-Islander	7 (0.9%)	3 (4.3%)	4 (0.6%)	0.046
Caucasian	700 (91.9%)	63 (91.3%)	637 (91.9%)	
Asian	41 (5.4%)	3 (4.3%)	38 (5.5%)	
Hispanic	4 (0.5%)	0 (0.0%)	4 (0.6%)	
Other	9 (1.2%)	0 (0.0%)	9 (1.3%)	
Middle Eastern	1 (0.1%)	0 (0.0%)	1 (0.1%)	
Disease duration at recruitment* (years)	6.58 (1.93-15.10)	9.19 (2.25-20.41)	6.38 (1.90-14.60)	0.028
Disease duration* to first RHC/or last review if no RHC (years)	13.00 (6.50-20.86)	14.37 (4.68-21.89)	12.83 (6.69-20.81)	0.955
Autoantibody profile (ever)				
ANA	786 (96.3%)	69 (97.2%)	717 (96.2%)	0.687
ANA nucleolar	188 (23.1%)	14 (19.7%)	174 (23.4%)	0.480
ANA homogenous	158 (19.4%)	9 (12.7%)	149 (20.1%)	0.132
ANA centromere	364 (44.7%)	35 (49.3%)	329 (44.2%)	0.411
ENA Scl-70	116 (14.3%)	7 (9.9%)	109 (14.7%)	0.265
RNA polymerase III	112 (16.2%)	7 (12.5%)	105 (16.5%)	0.435
ANCA	173 (22.2%)	17 (25.0%)	156 (21.9%)	0.562
MPO	33 (4.3%)	4 (6.1%)	29 (4.1%)	0.455

Variable	All patients (n=820)	PAH (n=71)	No PAH (n=749)	p value
PR3	33 (4.3%)	6 (9.1%)	27 (3.8%)	0.043
Anti-CCP	16 (3.8%)	1 (3.2%)	15 (3.9%)	0.858
Beta-2 glycoprotein	51 (32.3%)	4 (23.5%)	47 (33.3%)	0.414
Cardiolipin IgG	66 (40.5%)	5 (26.3%)	61 (42.4%)	0.181
Lupus anticoagulant	23 (3.1%)	3 (4.4%)	20 (3.0%)	0.519
Anti-ds DNA	93 (12.4%)	7 (10.3%)	86 (12.6%)	0.589
ENA Jo-1	2 (0.2%)	0 (0.0%)	2 (0.3%)	0.661
ENA La	17 (2.1%)	0 (0.0%)	17 (2.3%)	0.197
ENA U1RNP	67 (8.3%)	5 (7.0%)	62 (8.4%)	0.698
ENA Ro	72 (8.9%)	10 (14.1%)	62 (8.4%)	0.105
Anti-Scl/PM	20 (2.5%)	1 (1.4%)	19 (2.6%)	0.558
ENA Anti-Sm	23 (2.8%)	1 (1.4%)	22 (3.0%)	0.449
Rheumatoid factor	238 (30.0%)	27 (39.1%)	211 (29.1%)	0.082
ILD on HRCT ever (n=389)	227 (57.0%)	34 (58.6%)	193 (56.8%)	0.792
Highest severity of ILD on HRCT ever (n=214)				
Mild(<20%)	120 (56.1%)	12 (37.5%)	108 (59.3%)	0.012
Moderate(20-30%)	53 (24.8%)	8 (25.0%)	45 (24.7%)	
Severe(>30%)	41 (19.2%)	12 (37.5%)	29 (15.9%)	
Respiratory function test				
FVC %	95.50 (81.00-109.00)	81.00 (63.00-98.00)	96.00 (82.00-110.00)	<0.001
corrected DLCO %	73.00 (57.00-87.00)	43.57 (34.00-53.00)	75.26 (61.58-88.57)	<0.001
WHO functional class				
Class I	392 (52.3%)	1 (1.5%)	391 (57.3%)	<0.001
Class II	226 (30.2%)	13 (19.4%)	213 (31.2%)	
Class III	117 (15.6%)	45 (67.2%)	72 (10.6%)	
Class IV	14 (1.9%)	8 (11.9%)	6 (0.9%)	
Cardiovascular disease risk factors				

Variable	All patients (n=820)	PAH (n=71)	No PAH (n=749)	p value
Systemic hypertension	362 (44.4%)	34 (49.3%)	328 (43.9%)	0.391
Diabetes	64 (7.9%)	7 (10.3%)	57 (7.7%)	0.451
Angina/acute myocardial infarction	63 (7.7%)	8 (11.8%)	55 (7.4%)	0.193
Six minute walk distance (m)	450.00 (362.00-525.00)	339.00 (280.00-400.00)	475.00 (405.00-540.00)	<0.001
NT Pro BNP test value (ng/L)	131.17 (68.00-256.00)	403.31 (149.00-1641.50)	121.44 (67.00-229.52)	<0.001
Uric acid (mmol/L)	0.29 (0.24-0.35)	0.38 (0.29-0.46)	0.28 (0.24-0.34)	<0.001
Echocardiographic (TTE) parameters				
RA area (cm square)	15.00 (13.00-17.80)	18.00 (15.00-22.00)	15.00 (12.55-17.00)	<0.001
TR velocity (m/s)	2.50 (2.30-2.80)	3.35 (3.10-3.69)	2.48 (2.22-2.70)	<0.001
sPAP (mmHg)	31.00 (26.00-38.00)	51.50 (44.00-65.00)	30.00 (26.00-35.00)	<0.001
pericardial effusion (non-trivial)	31 (4.6%)	9 (12.9%)	22 (3.6%)	<0.001
Right heart catheterisation (RHC) parameters				
RAP (mmHg)	7.00 (5.00-10.00)	8.00 (6.00-11.00)	5.50 (4.00-9.00)	0.026
mPAP (mmHg)	28.00 (23.00-34.00)	30.00 (27.00-36.00)	21.00 (17.00-26.00)	<0.001
PAWP (mmHg)	11.00 (7.50-14.00)	11.00 (8.00-13.00)	13.00 (7.00-16.00)	0.153
PVR (WU)	2.98 (2.12-4.80)	3.46 (2.80-6.30)	1.80 (1.43-2.20)	<0.001
Missing PVR (n=71)	11 (15.5%)	11 (15.5%)	0 (.%)	.
Cardiac output (thermodilution) (L/min)	5.00 (4.02-6.02)	4.97 (3.83-6.12)	5.32 (4.50-5.97)	0.175
Cardiac index (L/min/m^2)	2.89 (2.32-3.17)	2.86 (2.03-3.10)	3.01 (2.60-3.19)	0.310
Heart rate (bpm)	73.00 (64.00-76.00)	73.00 (65.00-76.00)	72.50 (63.50-79.00)	0.903

Supplementary Table S1. contains the patient characteristics by PAH diagnosed on RHC. For categorical variables, a number (percentage) is presented with a p-value calculated by chi square test. For normally distributed continuous variables the mean (standard deviation) is presented with a p-value calculated by Student's two-sample T-test. For non-normally distributed continuous variables the median (interquartile range) is presented with a p-value calculated by Wilcoxon rank-sum test.

*Disease duration from first non-Raynaud's phenomenon SSc disease manifestation onset.

NTproBNP, N-terminal pro-brain type natriuretic peptide; RHC, right heart catheterisation; ANA, antinuclear antibody; ENA, extractble nuclear antigen; Scl-70, anti-Scl-70 antibody (anti-topoisomerase antibody); RNA, ribonucleic acid; ANCA, anti-neutrophil cytoplasmic antibody; MPO, anti-myeloperoxidase antibody; PR3, anti-proteinase 3 antibody; Anti-CCP, anti-cyclic citrinullated antibody; anti-dsDNA, anti-double stranded deoxyribonucleic acid antibody; ILD, interstitial lung disease; HRCT, high resolution computed tomography of the chest; FVC%, forced vital capacity % predicted; DLCO, diffusion capacity for carbon monoxide % predicted; WHO, World Health Organisation; RA, right atrial; TR, tricuspid regurgitant jet; sPAP, systolic pulmonary artery pressure; RAP, mean right atrial pressure; mPAP, mean pulmonary artery pressure; PAWP, pulmonary arterial wedge pressure; PVR, pulmonary vascular resistance.

Supplementary Table S2. Results of ROC curve analysis for NT-proBNP at different cut-points to identify PAH within 12 months (inclusion from 01 January 2016)

Variable	Sens	Spec	PPV	NPV	AUC	Cost
ASIG screening algorithm	100.00% (84.56% - 100.00%)	64.27% (61.67% - 66.80%)	4.28% (2.70% - 6.41%)	100.00% (99.58% - 100.00%)	0.82 (0.81 - 0.83)	$469.20
NT ProBNP above 210	77.27% (54.63% - 92.18%)	74.11% (71.94% - 76.19%)	3.77% (2.21% - 5.97%)	99.60% (99.07% - 99.87%)	0.76 (0.67 - 0.85)	$249.67
NT ProBNP above 122.9	90.91% (70.84% - 98.88%)	55.37% (52.95% - 57.77%)	2.60% (1.60% - 3.99%)	99.78% (99.23% - 99.97%)	0.73 (0.67 - 0.79)	$379.54
NT ProBNP above 186.6	81.82% (59.72% - 94.81%)	70.53% (68.28% - 72.70%)	3.52% (2.10% - 5.50%)	99.66% (99.14% - 99.91%)	0.76 (0.68 - 0.84)	$274.33

ROC, receiver operator characteristics; NT-proBNP, N-terminal pro-brain type natriuretic peptide; PAH, pulmonary arterial hypertension; Sens, sensitivity; Spec, specificity; PPV, positive predictive value; NPV, negative predictive value; AUC, area under the curve; cDLCO, corrected diffusion capacity for carbon monoxide % predicted; FVC, forced vital capacity % predicted

Supplementary Figures

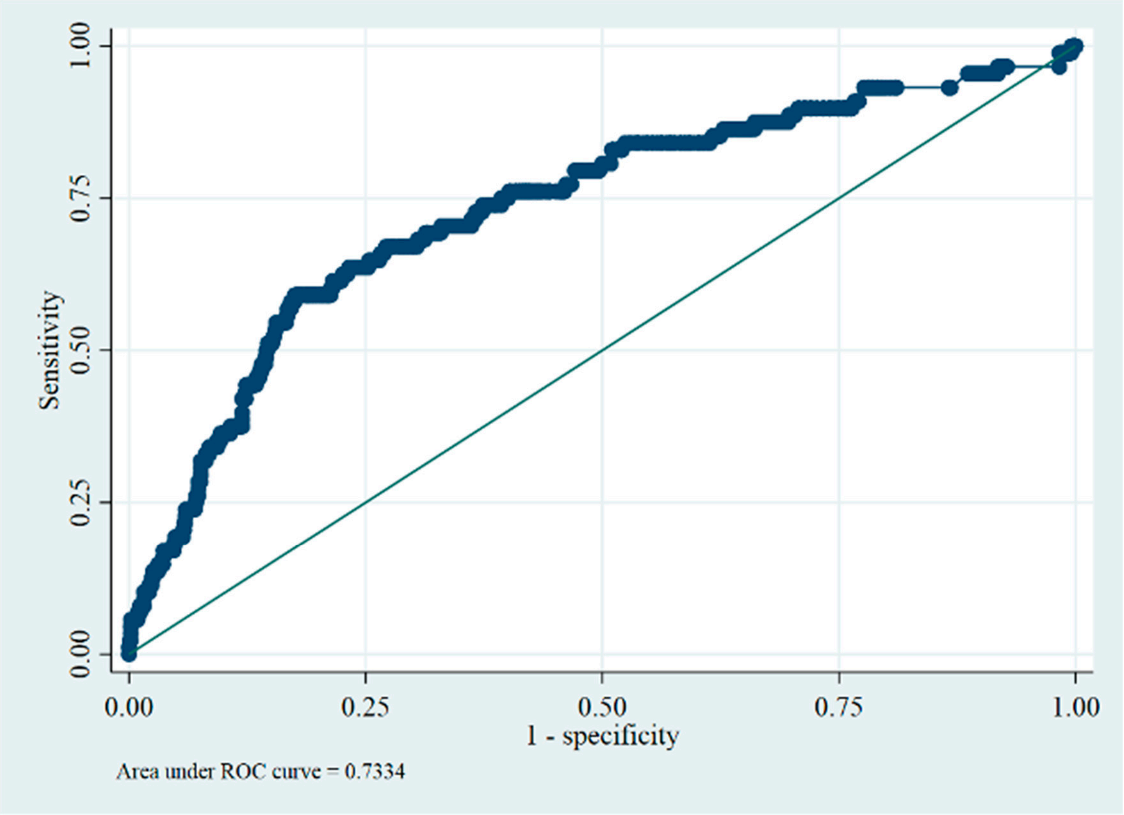

Supplementary Figure S1. Receiver operating characteristics (ROC) curve for serum NT-proBNP level to predict PAH diagnosis within 12 months (AUC = 0.7334)
